# Health-Training in Elementary Schools

**Published:** 1907-01-12

**Authors:** 


### The Hospital

A Journal of

The Medical Sciences and Hospital Administration.

Vol. XLI.—No. 1,061. SATURDAY, JANUARY 12, 1907.

### Health-Training in Elementary Schools.

Those aspects of the problem of elementary education which have for some time engaged public attention, are associated with controversies that lie altogether outside our province in these columns. We may, however, be permitted the remark, that the disputes of politicians and ecclesiastics, strenuous though they be, do not occupy the entire educational area, and that many departments of school life and work are happily beyond the reach of the prevailing controversy. Whether these are more or less important than the questions which excite the sects, we do not pretend to say, but it is certain that to an ever-increasing extent public opinion is learning to recognise that true education, even in its elementary developments, has for its aim, not merely to impart knowledge and to train the mind, but also to promote physical development and to form character. Hence the public, though wearied of the subject of elementary education, at least in its political relationships, may well wish to know how the actual work of the schools is being conducted, and to what extent this work is contributing to the promotion of manhood and moral worth in the nation. For, after all, whatever else is in dispute, this is certain, that unless these ends are attained, our national plan of education is a failure, and the future can bring nothing but anxiety and disaster.

Elementary educational arrangements, as a training and discipline for the rising generation, more especially in reference to physical health and well-being, have for some years been gaining the countenance and goodwill of the school authorities, and it is satisfactory to find, from a recent statement by Mr. A. J. Shepheard, Chairman of the Education Committee of the London County Council, that demands in this direction are fully recognised. The health of school children is obviously a question which controls all forms of educational activity. Thus, expert medical advice and control appear as essential parts of the educational problem, and this, more particularly, as the collection of large numbers of children together day after day means special risks to health, in reference more particularly to the spread of infectious disease. Delicate questions of responsibility, both to individual children and to the community generally, arise in this connection, and solutions for these questions can only be provided by competent hands. The care and supervision needed in the directions just indicated may be made of the first educational importance to the teachers, and through them to the children. Hence one reads with satisfaction that the head teachers of the schools in the metropolitan area are expected to give attention to such matters as the ventilation of the school buildings, the scrubbing of floors, and the cleansing of "offices." Children who come to school dirty are washed or, if necessary, are sent home for more searching methods of purification. In short, in these and other ways, a tone and influence are sustained which suggest day after day the value of cleanliness and fresh air as agents in the promotion of good health and the prevention of disease. Again, the cultivation of bodily vigour is now directly encouraged by the introduction into the curriculum of all elementary schools of systematised physical exercises. Here, also, as in many other modern educational developments, scientific method has largely superseded crude beginnings. To secure the interest of the mind is as much a part of these exercises as to strengthen the muscles and to promote bodily development; and games and athletics ought to be made instruments of mental and moral training as well as ends to physical well-being.

Detailed reference to the medical inspection of the children in the elementary schools may engage us on another occasion. It is sufficient here to remark that it has been made much more thorough and much more extensive than was even until recently the case, and though it is likely to raise some awkward questions, these ought not to be beyond the reach of statesmanship and goodwill. Public opinion will certainly view with increasing impatience attempts to drive children through an educational curriculum without taking adequate care to see that their bodily health fits them to take advantage of the process. Such attempts are cruel in themselves, and they display a complete failure to appreciate a true educational ideal. The L.C.C. are following a very different plan, and we believe they can justify their procedure. At all events, it is satisfactory to learn that, in spite of the din of controversy, the real educational work of the schools goes on peacefully and efficiently, and that those charged with the oversight and direction of it are fully alive to the relationships which necessarily exist between the *mens sana* and the *corpus sanum*.

